# Unusual Complication of Hemodialysis Cuffed Catheter Tunnel Infection and Unconventional Therapeutical Decision: A Report of Two Cases

**DOI:** 10.1155/2018/2405864

**Published:** 2018-11-19

**Authors:** Biser K. Borisov, Stela P. Linkova

**Affiliations:** Department of Nephrology and Dialysis, Medical University, Pleven, Bulgaria

## Abstract

**Introduction:**

Infectious complications are the most common chronic complications observed in patients undergoing hemodialysis with central venous catheters. However, despite the efforts of a large number of medical professionals, tunnel catheters are increasingly being used for hemodialysis in the everyday practice.

**Case Report:**

We describe two cases of an equal complication of a tunnel infection wherein the catheter becomes naked after self-rupture of the purulent secretion. We did not replace the tunnel catheter but applied a skin plastic by rotation flaps over the affected area, which proved to be sufficient. Six months after the intervention, the patients continue their hemodialysis treatment using the same cuffed catheters; the taken chemocultures do not give rise only to bacterial growth and skin plastic has been healed primary.

**Conclusions:**

The two cases described by us represent one treatment option, which may be discussed with reference to such specific two cases in practice.

## 1. Introduction

The incidence of catheter-related bacteraemia ranges between 0.6 and 6.5 episodes per 1000 catheter days [1, 2]. The clinical manifestations of catheter-associated infections are exit-site infection, tunnel infection, and catheter-related bloodstream infection.

The tunnel infection is defined by the Centers for Disease Control and Prevention (CDC) as a condition of tenderness, erythema, or site induration > 2 cm from the catheter site along the subcutaneous tract of a tunnel catheter in the mandatory absence of concomitant bloodstream infections (BSI) [2]. The modern clinical practice guidelines recommend removal of the catheter regarding these cases, incision and drainage if indicated, and 7–10 days of antibiotic therapy [3–9].

However, these recommendations are not relevant to cases where a cuffed catheter is the ultimate vascular access of the patient and is placed at the last possible position. In this report, we describe two cases of tunnel infection where the treatment was unconventional.

## 2. Case Report

We describe two cases of tunnel infection in a 54-year-old woman and a 57-year-old man, both Caucasian. The two patients had a history of exhausted vascular access due to the repeatedly constructed arteriovenous anastomoses and the preceding temporary and cuffed catheters for hemodialysis. In both cases, the catheters were placed more than nine months ago, and no evidence of infection and catheter dysfunction have been observed until now.

**Figure 1:**
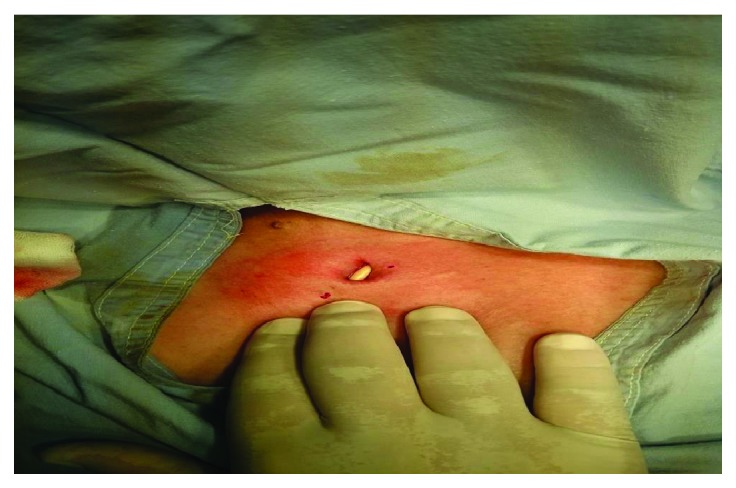
The “naked” part of the cuffed catheter.

The woman was transferred from peritoneal to hemodialysis therapy due to encapsulating peritonitis whereas the man underwent surgical intervention due to complications associated with chronic ulcerative colitis. In both cases, there was an established tunnel infection but the taken chemocultures did not detect the presence of bacteraemia and a treatment with vancomycin (empirically) was held along and locally with by applying povidone-iodine daily dressings. Two to three days after introducing the treatment, both patients had spontaneously evacuated the purulent secretions from the inflamed skin tunnel from which *Staphylococcus aureus* was isolated. After three intravenous applications of vancomycin of 15–30 mg/kg every 5 days in both patients, there was no evidence of infection in the described area. (Figure 1).

**Figure 2:**
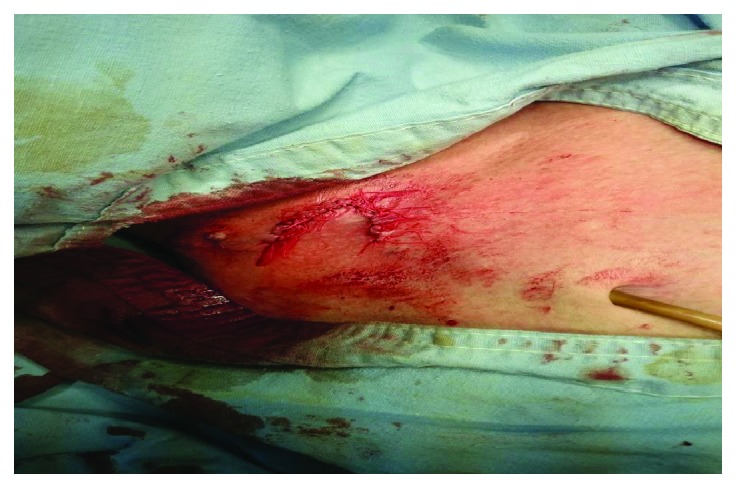
The “naked” zone is covered by rotation flap.

Following threefold cleaning of the operative field with povidone-iodine, local anaesthesia was applied with 1% lidocaine. The surrounding tissues were cut to 12–13 mm in healthy skin, including 8–10 mm below the catheter body. The area was washed with 1% chlorhexidine and the tissue defect on the catheter was enclosed with rotating flaps from the adjacent healthy area (Figure 2). The sutures from the surgical wound were removed on the seventh postoperative day. Prior to that, the patients were wearing daily dressing with povidone-iodine. Six months after the intervention, the patients continued their hemodialysis treatment with the same catheters and the chemocultures did not give rise to bacterial growth.

## 3. Discussion

The observed complications of central venous catheters for hemodialysis are divided into acute and chronic ones. The acute complications are directly attributable to the catheter insertion, while the chronic are related to their long-term use [10].

Catheter-related infections, such as catheter-related bloodstream infections (CRBSIs), exit-site infections, and tunnel infections, are common chronic complications among hemodialysis patients with vascular access central venous catheter [2, 5]. Infection is the second leading cause of death among patients on hemodialysis [5]. Depending on their combination, there are several management options—only antimicrobial therapy, antimicrobial therapy with catheter exchange, or catheter removal [7]. The clinical practice guidelines reflect the experience of many doctors over the years. They help us make the right decisions in standard situations. Considering the two cases described herein, the standard solution, according to the rules, was immediately to remove the cuffed catheter and insert a new catheter into another place. The replacement of a tunnel catheter over a guidewire is shown in cases of CRBSIs in the absence of a local infection engaging the subcutaneous tunnel. Actually, after the antibiotic treatment, if there is no evidence of a local infection, then changing the catheter over a guidewire is an option. It is a must to avoid the skin defect. In the two cases we described, we decided to treat without changing the cuffed catheter due to the presence of multiple adhesions in the subcutaneous tissues of previous catheterization that had affected the construction of a good new tunnel and to avoid a recurrent vascular trauma.

Catheter salvage should not be used in the following situations: *S. aureus*, *Pseudomonas*, and fungal infections; unresolved infectious symptoms 48 to 72 hours after initiation of antibiotics; metastatic complications; and concomitant tunnel infection [2, 3, 5, 7].

In the present cases, both the type and the place of vascular access were the last therapeutic options for these patients. The latter, along with the lack of BSI, were the reasons that this unconventional therapeutic modality was implemented.

## 4. Conclusion

Good clinical practice guidelines should be followed by doctors in daily practice while providing access for hemodialysis treatment. The two cases described by us represent only one treatment option under these specific conditions.
